# Health-related quality of life and patient-reported symptoms after postoperative proton beam radiotherapy of cervical and endometrial cancer: 2-year results of the prospective phase II APROVE-trial

**DOI:** 10.1186/s13014-023-02198-4

**Published:** 2023-01-09

**Authors:** Eva Meixner, Antje Wark, Tobias Forster, Fabian Weykamp, Kristin Lang, Laila König, Katja Lindel, Jan Tobias Oelmann-Avendano, Johannes Krisam, Andreas Schneeweiss, Malte Ellerbrock, Thomas Mielke, Juliane Hörner-Rieber, Klaus Herfarth, Jürgen Debus, Nathalie Arians

**Affiliations:** 1 Department of Radiation Oncology, Heidelberg University Hospital, Im Neuenheimer Feld 400, 69120 Heidelberg, Germany; 2 Heidelberg Institute of Radiation Oncology (HIRO), 69120 Heidelberg, Germany; 3 National Center for Tumor Diseases (NCT), Heidelberg, Germany; 4 Department of Radiation Oncology, Municipal Hospital Karlsruhe, 76133 Karlsruhe, Germany; 5 Department of Radiation Oncology, Göttingen University Hospital, 37075 Göttingen, Germany; 6 Institute for Medical Biometry and Informatics, University of Heidelberg, 69120 Heidelberg, Germany; 7 Department of Gynecology and Obstetrics, Heidelberg University Hospital, 69120 Heidelberg, Germany; 8 Heidelberg Ion-Beam Therapy Center (HIT), 450, 69120 Heidelberg, Germany; 9 Clinical Cooperation Unit Radiation Oncology, German Cancer Research Center (DKFZ), 69120 Heidelberg, Germany

**Keywords:** Uterine neoplasm, Radiochemotherapy, Gynecological cancer, Toxicity, Quality of life, Radiotherapy

## Abstract

**Introduction:**

The APROVE-trial investigated the tolerability of postoperative proton beam therapy in women with cervical or endometrial cancer. The present analysis evaluated the secondary endpoints of health-related quality of life (HRQOL) and patient-reported symptoms.

**Methods:**

25 patients were included in this prospective phase-II-trial and treated with postoperative radiotherapy using protons alone or in combination with chemotherapy. To attain general and gynecologic-specific HRQOL measures, the EORTC-QLQ-C30 questionnaires combined with -QLQ-CX24 for cervical and -QLQ-EN24 for endometrial cancer were assessed at baseline, at the end of RT and up to 2 years after radiotherapy. The results were compared to an age-matched norm reference population. Symptoms were assessed using Common Terminology Criteria for Adverse Events (CTCAE) and institutional patient-reported symptoms grading.

**Results:**

Scores regarding global health status were markedly impaired at baseline (mean: 58.0 ± 20.1) compared to reference population data, but significantly (*p* = 0.036) improved and evened out to comparable norm values 2 years after proton therapy (mean: 69.9 ± 19.3). Treatment caused acute and long-term worsening of pain (*p* = 0.048) and gastrointestinal symptoms (*p* = 0.016) for women with endometrial cancer, but no higher-grade CTCAE ≥ 3° toxicity was observed. Dosimetric evaluation of rectum, sigmoid, large and small bowel showed no correlation with the reported gastrointestinal symptoms. After 2 years, fatigue had significantly improved (*p* = 0.030), whereas patients with cervical cancer experienced more often lymphedema (*p* = 0.017). Scores for endometrial cancer pertaining to sexual activity (*p* = 0.048) and body image (*p* = 0.022) had improved post treatment; in the latter this effect persisted after 2 years.

**Conclusion:**

Proton beam therapy in the adjuvant setting was well tolerated with only low-grade side effects concerning gastrointestinal symptoms, lymphedema and pain. Overall quality of life was impaired at baseline, but patients were able to recover to values comparable to norm population 2 years after proton therapy. Larger studies are needed to confirm whether the benefit of proton therapy translates into a clinical effect. Sexual dysfunction remains an important issue.

*Trial registration*: The trial was registered at https://clinicaltrials.gov (ClinicalTrials.gov Identifier: NCT03184350, 09th June 2017).

**Supplementary Information:**

The online version contains supplementary material available at 10.1186/s13014-023-02198-4.

## Introduction

Cervical and endometrial cancer represent a considerable number of gynecologic neoplasms in women worldwide, with about 604,000 new cases of cervical and 417,000 new cases of uterine neoplasms diagnosed in 2020 [1]. In recent decades, the use of screening programs and optimized therapy options have led to a reduction in the associated mortality rate and the detection of these cancers in their earlier stages [2]. In addition to surgery, which represents the cornerstone of therapy in early-stage tumors, the use of postoperative radiotherapy or chemoradiation plays a crucial role in the treatment of women with high risk factors. However, the application of these concepts can be associated with a high rate of severe toxicity in about 7–15.3% of individuals in a trimodality treatment setting of surgery plus radiochemotherapy and in about 2–5.5% for surgery plus photon radiotherapy [3, 4]. The addition of chemotherapy has further been proven to have a negative long-term effect on toxicity and HRQOL [5]. Furthermore, 27% of women are at risk of developing at least partial vaginal narrowing and stenosis, leading to sexual dysfunction and impaired quality of life [6, 7].

Through the widespread use of conventional intensity-modulated radiation therapy techniques, treatment-related side effects caused by pelvic radiotherapy have been mitigated [8, 9] and currently represent the standard of care. Modern proton beam radiotherapy may be cost-intense, but it provides favorable physical characteristics resulting from a modulation of the Bragg-peak of protons with a maximum dose deposition within the target, a low entrance dose and a steep dose fall off behind. Further putative advantages of the use of proton instead of photon beams may include the delivery of fewer integral doses and increased radiobiological effects and biological tumor responses [10]. As a consequence, protons may significantly reduce the dose to surrounding organs at risk and improve therapy-related toxicity without diminishing local control. However, the historical literature has mostly explored this treatment in relation to intracranial, radioresistant oncologic lesions such as chondrosarcomas of the skull base [11, 12] and prospective clinical data for gynecological malignancies are lacking. While women with pelvic gynecologic cancers currently have no standard indication for proton beam therapy in Germany, planning comparison studies have proven that a significant benefit of protons is that the rate at which organs at risk are spared in gynecologic cancers is superior to the rates in other modern techniques of volumetric arc therapy or helical tomotherapy [13]. In case reports or study cohorts, only groups of about 7–11 women with gynecological malignancies were reported to be treated with proton beam therapy, but their dosimetric results, which revealed statistically significant lower doses to the bowel, bladder and bone marrow, are promising [14, 15].

The primary aim of this prospective APROVE (Adjuvant PROton therapy for cerVical and Endometrial cancer) phase II trial was to evaluate the clinical feasibility and safety of the use of postoperative pelvic proton beam therapy for women with gynecological malignancies and to assess the potential for the reduction in doses to the organs at risk. Here, we evaluated this translation into and impact on clinically detected effects on the secondary study endpoints (health-related quality of life (HQROL) outcomes and patient-reported symptoms).

## Materials and methods

### Patient population and study design

Between June 2017 and May 2020, 25 women with histologically confirmed cervical or endometrial cancer and an indication for postoperative radiotherapy were treated in this prospective single-center one-arm phase II APROVE trial at our Department of Radiation Oncology at the University Hospital of Heidelberg. A comprehensive study protocol and schedule has been previously reported. [16] The trial was registered at https://clinicaltrials.gov (ClinicalTrials.gov Identifier: NCT03184350, 09th June 2017). The study was approved by the local ethics committee (S-155/2016) and by an independent expert group from the German Society for Radiooncology (DEGRO). Treatment concepts were based on multidisciplinary tumor conference recommendations following international and institutional guidelines. All women received an upfront hysterectomy and were staged according to the FIGO 2018 staging system [17, 18]. Written informed consents was obtained from each participant prior to study inclusion.

#### Radiochemotherapy

Radiotherapy planning was performed with two computed tomography (CT) scans (full and empty bladder) with an immobilization ProSTEP (ITV, Innsbruck, Austria) device, which aided patient positioning, and a rectal balloon (in 17 of 25 patients). Target volume delineation of the clinical target volume (CTV) and organs-at-risk (OAR) was performed on CT planning image slices with 3 mm thicknesses on full-bladder CT scans and followed RTOG consensus guidelines for endometrial and cervical cancer [19], considering the position of the target and sparing volumes at different filling status of the bladder and considering several examinations and postoperative magnetic-resonance imaging whenever available. A planning target volume (PTV) margin of 0.5–0.7 cm accounting for internal target volume motions. Dose constraints to the organs at risk (at least the bladder, small and large bowel, sigmoid, rectum) were in accordance with common recommendations [20, 21].

Radiotherapy was delivered with a dose of 45–50.4 Gy [relative biological effectiveness (RBE)] in daily fractions of 5–6 × 1.8 Gy (RBE) per week using active raster scanning proton beam radiotherapy and posterior oblique beams with a median of two beams. Proton RBE was based on a fixed RBE relative to high-energy photons of 1.1. Daily orthogonal X-ray-based image guidance and regular CT scan treatment verification was applied. A high-dose-rate brachytherapy boost of 10 Gy using Iridium-192 in two fractions was applied to the upper two thirds of the vaginal cuff and at 5 mm depth to the vaginal mucosa with an intracavitary single vaginal cylinder after the end of proton beam therapy.

Chemotherapy was not a mandatory part of the study trial protocol. If indicated, simultaneous weekly cisplatin (40 mg/m^2^) for patients with cervical cancer, with a total target dose of 200–240 mg/m^2^ in five to six cycles was administered according to institutional guidelines. Women with endometrial cancer and an indication for postoperative chemotherapy received six cycles of sequential chemotherapy with carboplatin (AUC5) and paclitaxel (175 mg/m^2^).

### Study outcome measures

The primary objective of the APROVE trial was to assess the feasibility, safety, and treatment tolerability of postoperative pelvic proton beam therapy, which was defined as the lack of any CTCAE grade ≥ 3 gastrointestinal or urogenital toxicity or early therapy cessation. The detailed results relating to these parameters are presented separately. The current analysis describes the secondary endpoints of HRQOL and patient-reported symptoms at baseline and up to 2 years after proton beam therapy.

Patient-reported HRQOL was assessed using the EORTC QLQ-C30 (version 3.0) questionnaire with scoring of the global health status (in which higher scores represent higher HRQOLs), a functional scale of five items (physical, role, cognitive, emotional, and social; higher scores represent higher levels of functioning) and a symptom scale of nine items (fatigue, nausea and vomiting, pain, dyspnea, insomnia, appetite loss, constipation, diarrhea and financial difficulties; in which higher scores represent higher levels of problems) [22]. Additionally, an EORTC QLQ-CX24 module for cervical cancer [23] and EORTC QLQ-EN24 for endometrial cancer [24] were applied. Symptom and function items were rated from 1 (not at all) to 4 (very much), while the overall quality of life was scored from 1 (very poor) to 7 (excellent). The questionnaires were completed at baseline, at the end of radiotherapy and at 6 weeks and 3, 6, 9, 12 and 24 months after radiotherapy, except for patients who experienced local or distant progressive oncologic disease. Participants were censored at the time of disease progression. In addition to the EORTC-QLQ questionnaires, the incidences and grading of symptoms for each timepoint were assessed with an institutional grading system of patient-reported symptoms for: vaginal bleeding (none, mild, moderate, transfusion indicated), pain in general (none, mild, moderate, severe), stool frequency (2–3 times per week, once daily, 2–3 times per day, > 3 times per day), fecal incontinence (none, occasional, daily, severe), urinary incontinence (none, occasional, spontaneous, intervention indicated), urinary frequency (none, mild, moderate, severe), nocturia (none, 1–3 times per night, 4–6 times per night, > 6 times per night)). Further, an assessment of acute (≤ 90 days) and late (> 90 days) toxicity symptoms graded according to the Common Terminology Criteria for Adverse Events (CTCAE, version 4.0) was performed.

### Statistical analysis

Scores were linearly transformed and calculated to the standardized range of 0 to 100 using single data or multiple items from associated questions according to the scoring manual of the EORTC Quality of Life group [25]. Some items that assessed sexuality health issues were optional as they required the individual to be sexually active in order to answer. Scores were only calculated from eligible patients. Missing data and their extent were documented as described in the EORTC QLQ manual. If single-item measures or more than half of the values for multiple items were missing, the corresponding scale score was classified as missing; otherwise, the values for missing items were assumed to be equal to the average of those items that were present. The questionnaire return rates are provided in the corresponding tables.

As previously published studies have recommended, a score change of < 10 points was defined as stable, while a change of ≥ 10 points was set as clinically significant to define an improvement or deterioration of symptoms and functions [26, 27]. Guidelines for the interpretation of clinical relevance for the EORTC QLQ-C30 results were applied to categorize trivial, small, medium and large importance levels [28]. Furthermore, a comparison with age-matched normative means in the German population for the EORTC QLQ-C30 scales was performed [29].

The Mann–Whitney-U and *t* tests were applied to compare continuous variables, and the Pearson Chi-Square tests were used to assess categorical data. Data was categorized into age groups (< 60 vs. ≥ 60 years), Karnofsky performance score (≤ 80 vs. > 80), BMIs (normal vs. abnormal), radiation fields (extended vs. pelvic), simultaneous chemotherapy (yes vs. no), sequential chemotherapy (before vs. after), type of surgery (total abdominal hysterectomy vs. total laparoscopic hysterectomy), lymph node dissection (yes vs. no), FIGO stage (1/2 vs. 3/4) or primary tumor (endometrial vs. cervical cancer).

Further, linear regression was used to analyze dosimetric factors [mean, maximum, V(10 Gy), V(15 Gy), V(20 Gy), V(30 Gy) and V(40 Gy)] associated with HRQOL symptom scales at the end and 2 years after radiotherapy, except for the features: financial difficulties, body image and sexual issues, hair loss and tase change. A *p* value of less than 0.05 was considered statistically significant. Detailed descriptive data and scores are presented in tables. Data were compared between the baseline values and values at the end of RT, as well as between values at baseline and 24 months after RT. For statistical calculations IBM statistical software SPSS (Armonk, NY, USA, version 28) was used.

## Results

### Study population

Between June 2017 and May 2020, 26 patients were included in the APROVE trial; due to a missing cost coverage, there was a drop-off of one patient prior to the planning of RT. The median follow-up time after the end of proton beam therapy was 25.1 (range: 20.2–50.3) months. Within this, seven patients experienced progressive disease. The total cohort consisted of 25 women with a median age of 62 years and included 17 (68.0%) endometrial and 8 (32.0%) cervical cancer patients.

Upfront surgery was performed as hysterectomies with bilateral salpingo-oophorectomies; all but one patient (cN0) received lymph node dissection. Radiotherapy was administered after a median time of 85 days after surgery. The patients received a median dose of 50.4 (range: 45.0–50.4) Gy in 25–28 fractions followed by brachytherapy with a median dose of 10 Gy in 2 fractions. A nodal boost was not applied, postoperative imaging did not detect residual nodal metastases. All patients completed proton beam therapy as planned. All but one participant received brachytherapy after the completion of proton beam therapy; for one woman there was an overlap of 1 day at the penultimate day of proton therapy. Chemotherapy was administered to seven women with cervical cancer with simultaneous weekly cisplatin (40 mg/m^2^) with a median of five cycles and with sequential carboplatin and paclitaxel to 13 women with endometrial cancer with a median of six cycles. Detailed patient and treatment characteristics are presented in Table 1 . An additional table file shows the dose characteristics to organs-at-risk and planning target volume in more detail [see Additional file 1].

**Table 1:** Patient and treatment characteristics

Characteristics	Values (range or percentage)
Median age (years)	62 (33–78)
Age (years)	
31–40	3 (12.0%)
41–50	1 (4.0%)
51–60	8 (32.0%)
61–70	10 (40.0%)
71–80	3 (12.0%)
FIGO stage (2018)	
1	15 (60.0%)
2	2 (8.0%)
3	7 (28.0%)
4	1 (4.0%)
Median body mass index prior to RT (kg/m^2^)	25.0 (18.2–41.6)
Median Karnofsky performance score prior to RT (%)	90 (70–100)
Type of surgery	
TAH-BSO with LND	18 (72.0%)
TAH-BSO without LND	1 (4.0%)
TLH-BSO with LND	6 (24.0%)
Prior surgical lymph node dissection	
Yes	24 (96.0%)
No	1 (4.0%)
Median time from surgery to RT start	85 (36–232)
Chemotherapy	
Simultaneous (cisplatin weekly)	7 (28.0%)
Prior to radiotherapy (carboplatin/paclitaxel)	8 (32.0%)
After radiotherapy (carboplatin/paclitaxel)	5 (20.0%)
No chemotherapy	5 (20.0%)
Median RT treatment time (days)	43 (33–51)
Proton beam therapy dose	
45.0 GyRBE	9 (36.0%)
50.4 GyRBE	16 (64.0%)
Extended field radiation	
Including para-aortic region	1 (4.0%)
No extended para-aortic field	24 (96.0%)

*BSO* bilateral salpingo-oophorectomy, *FIGO* International Federation of Obstetrics and Gynecology, *LNE* lymph node dissection, *RBE* relative biological effectiveness, *RT* radiotherapy, *TAH* total abdominal hysterectomy, *TLH* total laparoscopic hysterectomy

### EORTC QLQ-C30: functional scales and global health status

**Fig. 1:**
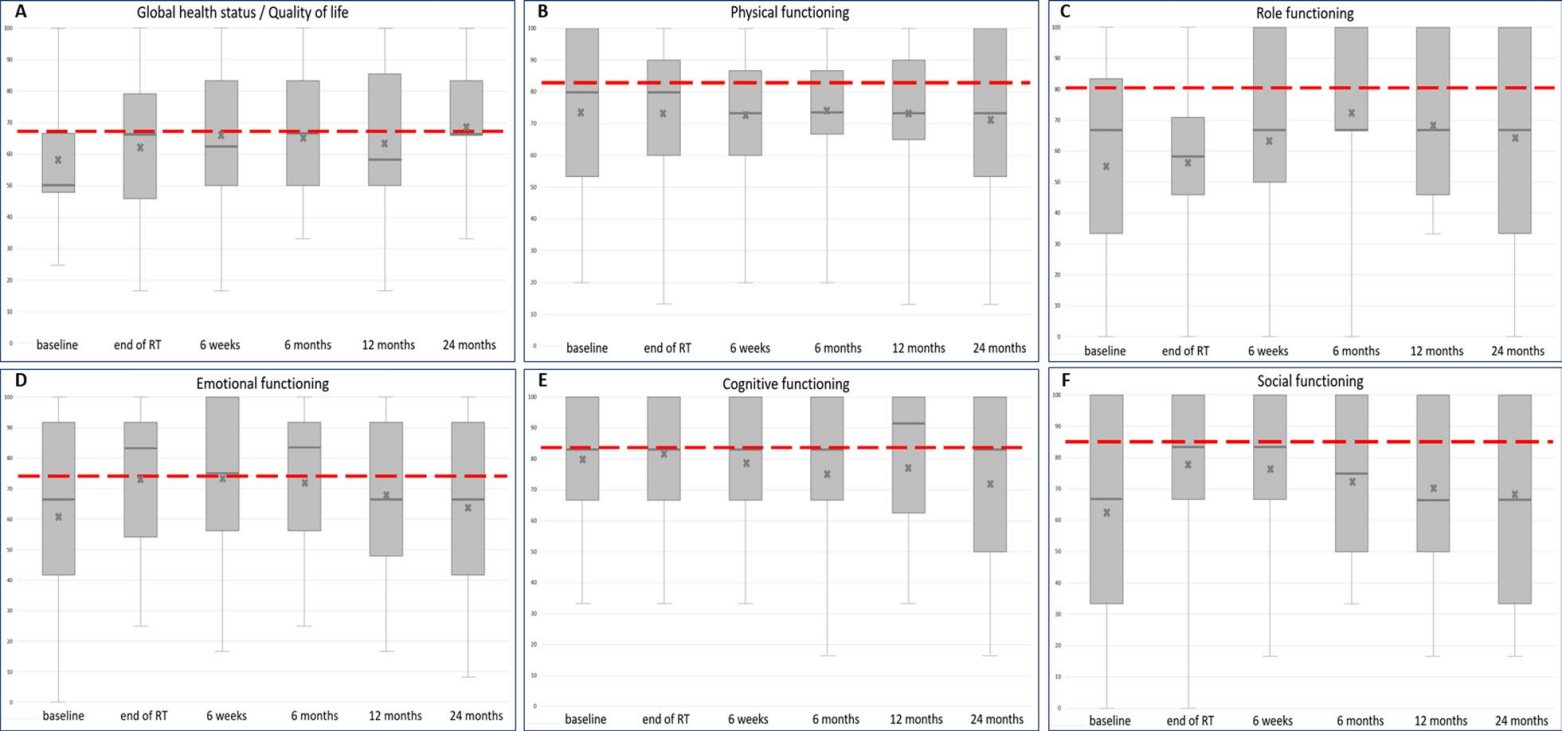
Global health status (**A**) and functional subscales (**B**–**F**) of the EORTC QLQ-C30 with boxplots for each time point of assessment (including minimum and maximum, median, mean = x, first and third quartiles) and mean values of German norm population (red line) [29]

Detailed results regarding the EORTC QLQ-C30 are presented in Fig. 1 for the global health status (A) and functional subscales (B–F) and are further summarized for all timepoints in Table 2.

**Table 2:** Mean quality of life scores (EORTC QLQ-C30, -CX24 and -EN24) with standard deviations (SD) at each time point

Score	Baseline		End of RT			6 weeks after RT		3 months after RT		6 months after RT		1 year after RT		2 years after RT		
	N	Mean (± SD)	N	Mean (± SD)	*p* value	N	Mean (± SD)	N	Mean (± SD)	N	Mean (± SD)	N	Mean (± SD)	N	Mean (± SD)	*p* value
*EORTC QLQ-C30*																
Global health status/QoL	24	58.0 (± 20.1)	23	61.6 (± 21.2)	0.573	24	66.0 (± 20.4)	23	66.7 (± 22.0)	24	65.6 (± 19.1)	24	62.8 (± 24.2)	18	69.9 (± 19.3)	0.036^b^
Functional scales (higher scores indicate a better QoL)																
Physical functioning	25	73.9 (± 24.1)	24	73.3 (± 23.9)	0.472	25	72.3 (± 21.0)	23	74.5 (± 21.7)	25	73.6 (± 21.3)	24	73.3 (± 21.8)	17	71.8 (± 26.3)	0.095
Role functioning	25	55.3 (± 34.2)	24	56.9 (± 31.1)	0.422	25	63.3 (± 28.3)	23	63.8 (± 30.9)	25	72.0 (± 27.4)	24	67.4 (± 25.7)	17	64.7 (± 29.6)	0.174
Emotional functioning	25	60.7 (± 30.5)	23	73.2 (± 24.2)	0.004	24	73.6 (± 24.0)	23	68.1 (± 21.7)	24	72.9 (± 24.0)	24	67.0 (± 26.8)	17	63.7 (± 27.9)	0.154
Cognitive functioning	25	80.0 (± 24.0)	23	82.6 (± 21.1)	0.500	24	79.2 (± 22.2)	23	79.0 (± 23.2)	24	76.4 (± 26.3)	24	77.8 (± 24.8)	17	71.6 (± 31.2)	0.335
Social functioning	25	62.0 (± 33.5)	23	78.3 (± 24.8)	0.008^c^	24	76.4 (± 25.0)	23	75.4 (± 26.9)	24	72.2 (± 25.8)	24	70.1 (± 25.9)	17	68.6 (± 29.1)	0.094
Symptom scales (higher scores indicate more problems)																
Fatigue	25	48.4 (± 26.6)	24	50.5 (± 27.8)	0.363	25	43.6 (± 28.1)	23	38.2 (± 27.8)	25	37.3 (± 26.6)	24	40.3 (± 26.6)	17	35.9 (± 26.0)	0.030^a^
Nausea and vomiting	25	8.0 (± 18.9)	24	15.3 (± 20.4)	0.142	25	6.0 (± 10.4)	23	8.0 (± 16.2)	25	5.3 (± 11.3)	24	11.8 (± 21.8)	17	5.9 (± 11.3)	0.402
Pain	25	34.0 (± 31.1)	24	34.0 (± 26.6)	0.453	25	32.0 (± 27.9)	23	35.5 (± 29.2)	25	33.3 (± 33.3)	24	38.2 (± 33.5)	17	30.4 (± 29.3)	0.294
Dyspnoea	23	23.2 (± 28.5)	24	34.7 (± 31.1)	0.035^b^	25	34.7 (± 25.8)	23	24.6 (± 22.5)	25	29.3 (± 25.5)	24	33.3 (± 25.5)	17	25.5 (± 29.2)	0.135
Insomnia	25	53.3 (± 35.3)	24	41.7 (± 35.0)	0.108	25	40.0 (± 35.3)	23	42.0 (± 22.5)	25	44.0 (± 34.9)	24	48.6 (± 31.9)	17	52.9 (± 39.7)	0.266
Appetite loss	25	22.7 (± 30.9)	23	24.6 (± 29.8)	0.266	25	12.0 (± 18.6)	23	11.6 (± 25.3)	25	12.0 (± 20.8)	24	16.7 (± 25.5)	16	12.5 (± 20.0)	0.191
Constipation	25	37.3 (± 36.9)	24	33.3 (± 37.3)	0.203	24	25.0 (± 30.8)	23	27.5 (± 30.5)	23	36.2 (± 33.9)	24	29.2 (± 36.4)	17	29.4 (± 32.1)	0.430
Diarrhoe	25	13.3 (± 23.1)	22	22.7 (± 30.8)	0.105	24	11.1 (± 15.7)	23	14.5 (± 21.6)	24	11.1 (± 15.7)	24	15.3 (± 28.8)	17	13.7 (± 20.0)	0.254
Financial difficulties	25	14.7 (± 25.1)	22	21.7 (± 28.8)	0.154	24	23.6 (± 28.0)	21	23.8 (± 31.1)	24	22.2 (± 28.3)	24	22.2 (± 29.9)	17	21.6 (± 30.1)	0.107
*EORTC QLQ-CX24*																
Functional scales (higher scores indicate a better QoL)																
Body image	8	73.6 (± 24.2)	7	81.0 (± 22.8)	0.443	8	65.3 (± 27.5)	8	70.8 (± 20.7)	8	69.4 (± 22.0)	6	75.9 (± 23.5)	5	53.3 (± 27.6)	0.179
Sexual activity	8	8.3 (± 22.0)	7	25.0 (± 25.0)	0.178	7	14.3 (± 16.5)	7	19.0 (± 24.3)	5	20.0 (± 26.7)	6	27.8 (± 22.9)	4	6.7 (± 13.3)	0.352
Sexual enjoyment	1	100.0 (± 0.0)	1	47.6 (± 35.0)	NA	2	33.3 (± 33.3)	3	66.7 (± 27.2)	2	83.3 (± 25.2)	4	66.7 (± 23.6)	1	100.0 (± 0.0)	NA
Sexual/vaginal functioning	1	58.3 (± 0.0)	1	75.0 (± 0.0)	0.104	2	50.0 (± 25.0)	3	47.2 (± 25.8)	2	54.2 (± 29.2)	4	56.3 (± 23.8)	1	83.3 (± 0.0)	0.305
Symptom scales (higher scores indicate more problems)																
Symptom experience	8	19.7 (± 12.9)	7	23.8 (± 9.9)	0.188	7	22.1 (± 13.4)	7	17.3 (± 15.5)	8	16.7 (± 13.8)	6	11.4 (± 7.2)	4	20.5 (± 8.7)	0.277
Lymphoedema	8	16.7 (± 23.6)	7	33.3 (± 39.8)	0.267	8	60.4 (± 37.2)	8	41.7 (± 36.3)	8	37.5 (± 30.9)	6	25.0 (± 14.4)	5	53.3 (± 26.7)	0.017
Peripheral neuropathy	8	8.3 (± 14.4)	7	11.1 (± 24.8)	0.499	8	4.2 (± 11.0)	8	16.7 (± 23.6)	8	20.8 (± 23.2)	6	16.7 (± 28.9)	5	0.0 (± 0.0)	0.284
Menopausal symptoms	8	37.5 (± 30.9)	7	47.6 (± 39.3)	0.284	7	19.0 (± 35.0)	7	42.9 (± 38.7)	8	50.0 (± 28.9)	6	41.7 (± 27.6)	4	16.7 (± 28.9)	0.359
Sexual worry	8	58.3 (± 36.3)	7	23.8 (± 34.3)	0.055	7	33.3 (± 39.8)	6	44.4 (± 45.8)	6	38.9 (± 40.4)	5	33.3 (± 40.8)	2	50.0 (± 50.0)	0.397
*EORTC QLQ-EN24*																
Functional scales (higher scores indicate a better QoL)																
Sexual interest	16	20.8 (± 28.6)	15	28.9 (± 29.5)	0.104	17	35.4 (± 27.6)	14	35.7 (± 26.6)	16	25.0 (± 25.0)	13	30.8 (± 30.6)	8	22.2 (± 15.7)	0.305
Sexual activity	15	13.3 (± 20.4)	16	25.0 (± 25.0)	0.048	15	26.7 (± 24.9)	13	25.6 (± 26.6)	16	22.9 (± 22.7)	14	26.2 (± 28.7)	8	12.5 (± 23.2)	0.086
Sexual enjoyment	4	58.3 (± 27.6)	7	47.6 (± 35.0)	NA	9	66.7 (± 22.2)	7	71.4 (± 21.3)	7	66.7 (± 25.2)	6	83.3 (± 25.5)	3	55.6 (± 31.4)	NA
Symptom scales (higher scores indicate more problems)																
Lymphoedema	17	44.1 (± 37.0)	17	31.4 (± 29.6)	0.069	17	38.2 (± 33.7)	15	40.0 (± 28.4)	17	43.1 (± 33.4)	15	41.1 (± 34.4)	10	43.3 (± 34.3)	0.069
Urological symptoms	17	19.1 (± 21.0)	16	25.5. (± 24.6)	0.175	17	25.5 (± 26.0)	15	23.9 (± 19.0)	16	17.7. (± 15.0)	14	22.6 (± 21.5)	8	37.5 (± 29.2)	0.175
Gastrointestinal symptoms	17	18.8 (± 9.8)	16	29.6 (± 21.6)	0.016	17	20.0 (± 13.1)	15	25.3 (± 12.7)	16	23.8 (± 18.1)	14	22.4 (± 19.3)	8	27.5 (± 9.1)	0.016
Poor body image	16	28.1 (± 30.5)	17	17.6 (± 21.7)	0.022	16	16.7 (± 25.7)	15	17.8 (± 23.1)	17	17.6 (± 23.9)	15	9.3 (± 16.9)	10	18.3 (± 20.3)	0.022
Sexual/vaginal problems	5	26.7 (± 24.9)	8	19.8 (± 19.7)	0.121	10	34.4 (± 21.3)	8	34.7 (± 31.6)	9	33.3 (± 25.7)	8	34.7 (± 29.6)	3	33.3 (± 32.7)	0.121
Pain in back and pelvis	17	21.6 (± 27.9)	16	29.2 (± 23.2)	0.048	17	35.3 (± 31.2)	14	38.1 (± 27.8)	16	43.8 (± 30.5)	14	40.5 (± 33.8)	8	33.3 (± 33.3)	0.048
Tingling/numbness	17	51.0 (± 38.1)	17	41.2 (± 38.8)	0.144	16	47.9 (± 40.8)	15	46.7 (± 36.1)	17	45.1 (± 39.5)	14	50.0 (± 37.3)	9	44.4 (± 44.4)	0.144
Muscular pain	16	41.7 (± 46.1)	16	27.1 (± 26.9)	0.075	16	39.6 (± 37.7)	15	33.3 (± 33.2)	15	41.7 (± 36.3)	14	40.5 (± 33.8)	7	47.6 (± 35.0)	0.075
Hair loss	17	39.2 (± 46.1)	15	20.0 (± 34.0)	0.215	17	11.8 (± 22.7)	15	31.1 (± 39.4)	15	18.8 (± 33.3)	14	4.8 (± 11.7)	8	33.3 (± 37.3)	0.215
Taste change	17	19.6 (± 29.6)	16	8.3 (± 18.6)	0.052	17	7.8 (± 18.2)	15	24.4 (± 35.4)	15	16.7 (± 31.2)	14	14.3 (± 30.1)	8	8.3 (± 14.4)	0.052

A *p* value of less than 0.05 was considered statistically significant for comparison of values to baseline

*N* number of available data, *NA* not assessable, *QoL* quality of life, *RT* radiotherapy

^a^Clinically relevant small difference

^b^Clinically relevant medium difference

^c^Clinically relevant large difference

Scores relating to the global health status/overall quality of life significantly improved 2 years after the end of radiotherapy from 58.0 (± 20.1) at baseline to 69.9 (± 19.3) (*p* = 0.036, 95%-Confidence Interval (CI): − 23.7 to 1.1) with medium clinical relevance. At the end of radiotherapy, a clinically large effect of the improvement of social functioning (*p* = 0.008, 95%-CI − 2.63 to 2.60) could be observed. Emotional functioning improved at the end of RT (*p* = 0.004, 95%-CI − 15.56 to − 2.54). Acute physical, role and cognitive functioning remained unchanged. No long-term significant differences were observed in physical, role, emotional, cognitive or social functioning after 2 years compared to the baseline values.

### EORTC-symptom scales and patient-reported symptoms

Except for increased rates of dyspnea (*p* = 0.035, 95%-CI 5.5 to − 22.1, medium clinical significance) at the end of RT and long-term improvements in the rates of fatigue (*p* = 0.030, 95%-CI − 20.1 to 6.2, small clinical difference) after 2 years, the EORTC QLQ-C30 did not detect any significant changes in symptom scales.

The EORTC QLQ-CX24 symptom scales observed significantly higher levels of lymphedema (*p* = 0.017, 95%-CI − 62.6 to − 4.0) in women with cervical cancer after 2 years, with a mean score of 53.3 compared to 16.7 at baseline. Women with endometrial cancer significantly experienced the acute worsening of gastrointestinal symptoms (*p* = 0.016, 95%-CI − 20.6 to − 1.1) and back and pelvic pain (*p* = 0.048, 95%-CI − 22.9 to 2.1) at the end of RT, which was persistent after 2 years. Detailed results regarding the EORTC QLQ-CX24 and -EN2 are summarized in Table 2.

No significant correlations between the doses to organs at risk (bladder, rectum, sigmoid, small bowel and large bowel) for Dmax, Dmean, V(10 Gy), V(15 Gy), V(20 Gy), V(30 Gy) and V(40 Gy) and symptom scale features at the end of RT and after 24 months could be found.

**Fig. 2:**
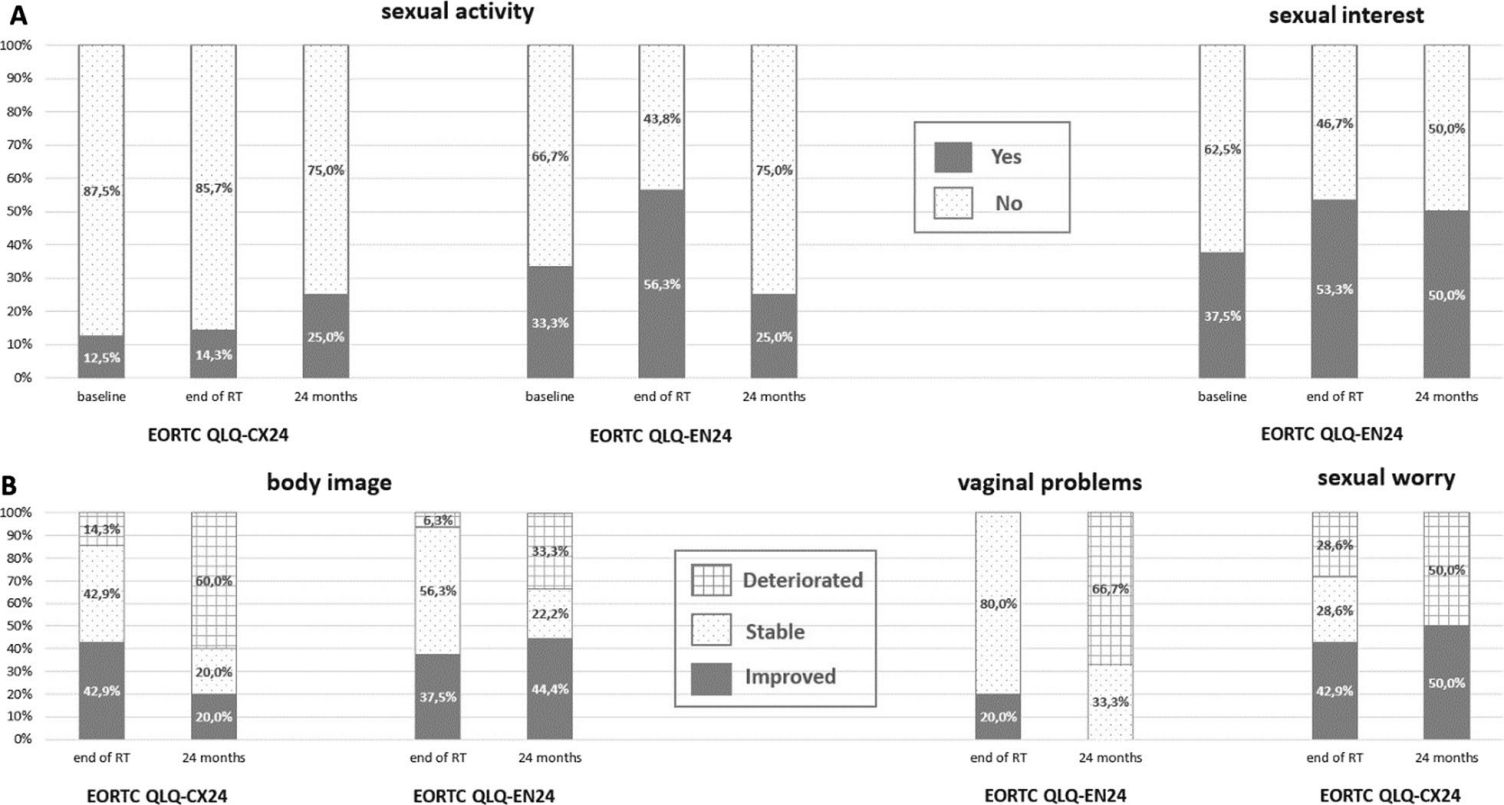
**A** Rates for the presence of sexual activity and sexual interest at baseline, at the end of RT and after 2 years. **B** Clinical changes of the body image, vaginal problems and sexual worry at the end of RT and after 2 years compared to baseline values

Results related to sexual (dys)function from the EORTC QLQ-CX24 and -EN24 subscales are presented in Fig. 2.

Sexual activity at baseline was only present in 12.5% of patients with cervical cancer and 33.3% of women with endometrial cancer and significantly increased in the latter at the end of RT (*p* = 0.048, 95%-CI − 24.5 to 2.3) (Fig. 2A) without any significant long-term effects. Sexual interest scores did not change significantly during the course of the observation. There was a trend that showed long-term deteriorated body image in 60% of the women with cervical cancer (Fig. 2B), while the patients’ body image significantly improved in women with endometrial cancer at the end of RT (*p* = 0.022, 95%-CI 0.36–22.6) and after 2 years (*p* = 0.022, 95%-CI 0.36–22.6). Compared to the baseline values patients with endometrial cancer reported improved or at least stable self-perception in 93.8% and 66.6% of the patients at therapy completion and after 2 years, respectively (Fig. 2B). Long-term increased vaginal problems in endometrial cancer were present, but without statistical significance; no significant effect on sexual worries could be assessed (Fig. 2B). Due to low response rates for questions regarding sexual enjoyment, no statistical analysis was performed.

Overall, the change in scores regarding global health, functional and symptom scales from baseline to 24 months did not significantly correlate with any of the patient and treatment characteristics among age groups (< 60 vs. ≥ 60 years), Karnofsky performance score (≤ 80 vs. > 80), BMIs (normal vs. abnormal), radiation fields (extended vs. pelvic), simultaneous chemotherapy (yes vs. no), sequential chemotherapy (before vs. after), type of surgery (total abdominal hysterectomy vs. total laparoscopic hysterectomy), lymph node dissection (yes vs. no), FIGO stage (1/2 vs. 3/4) or primary tumor (endometrial vs. cervical cancer).

In addition to health-related quality of life assessments using EORTC questionnaires, the incidences and grading of symptoms for each timepoint were assessed with an institutional grading system of patient-reported symptoms as well as an assessment of symptoms according to CTCAE version 4. According to the latter, no higher-grade CTCAE ≥ 3° gastrointestinal or genitourinary symptoms, as defined as primary study objectives, were reported.

**Fig. 3:**
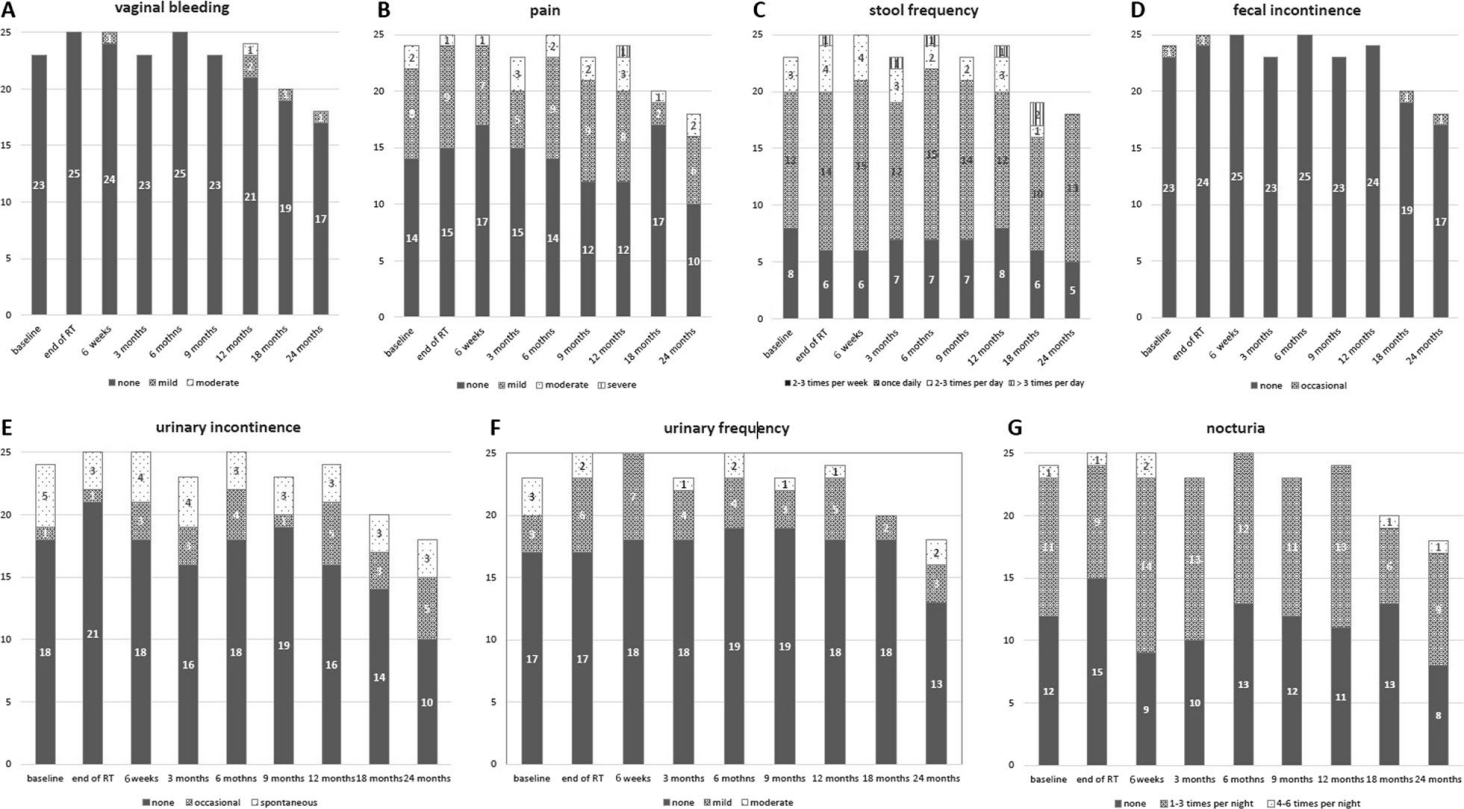
Incidence and grading of patient-reported symptoms for each timepoint for vaginal bleeding (**A**), pain (**B**), stool frequency (**C**), fecal incontinence (**D**), urinary incontinence (**E**), urinary frequency (**F**) and nocturia (**G**). Grading in Fig. 3 follows the institutional study grading system as defined in the picture and the methods section

Patient-reported symptoms in more detail are presented in Fig. 3 for vaginal bleeding (A), pain (B), stool frequency (C), fecal incontinence (D), urinary incontinence (E), urinary frequency (F) and nocturia (G). One patient experienced grade III° pain at the 12-month assessment, retrospectively caused by pain from a bone metastasis, that was diagnosed 1 month later. No significant correlation between patient-reported symptoms and significantly deteriorated long-term declines in pain and gastrointestinal symptoms in patients with endometrial cancer were found.

### Comparison with normative population

To a small clinical extent, scores regarding global health status (*p* = 0.042, 95%-CI 0.34–17.7) and physical functioning (*p* = 0.041, 95%-CI − 1.2 to 19.1) were both lower in participating women than in the age-matched normative German population at baseline, but global health status approached normal values 2 years after proton beam therapy. Long-term physical functioning (*p* = 0.035, small clinical significance), role functioning (*p* = 0.035, 95%-CI 0.4–31.8, medium clinical significance) and social functioning (*p* = 0.026, 95%-CI 0.8–31.6, large clinical significance) scores remained significantly lower than in the age-matched norm population. The detailed results are reported in Table 3.

**Table 3:** Results of mean quality of life scores (EORTC QLQ-C30) at baseline and after 2 years and mean values of German norm population [28]

Score	Norm population	Baseline			2 years after RT		
	Mean (± SD)	N	Mean (± SD)	*p* value	N	Mean (± SD)	*p* value
*EORTC QLQ-C30*							
Global health status/QoL	67.0 (± 21.8)	24	58.0 (± 20.1)	0.042^a^	18	69.9 (± 19.3)	0.353
Functional scales (higher scores indicate a better QoL)							
Physical functioning	82.8 (± 21.2)	25	73.9 (± 24.1)	0.041^a^	17	71.8 (± 26.3)	0.035^a^
Role functioning	80.8 (± 27.2)	25	55.3 (± 34.2)	< 0.001^b^	17	64.7 (± 29.6)	0.035^b^
Emotional functioning	73.9 (± 24.7)	25	60.7 (± 30.5)	0.044	17	63.7 (± 27.9)	0.082
Cognitive functioning	83.9 (± 22.7)	25	80.0 (± 24.0)	0.434	17	71.6 (± 31.2)	0.137
Social functioning	84.8 (± 25.5)	25	62.0 (± 33.5)	0.003^c^	17	68.6 (± 29.1)	0.026^c^
Symptom scales (higher scores indicate more problems)							
Fatigue	31.5 (± 27.2)	25	48.4 (± 26.6)	0.003^b^	17	35.9 (± 26.0)	0.364
Nausea and vomiting	6.0 (± 17.2)	25	8.0 (± 18.9)	0.406	17	5.9 (± 11.3)	0.786
Pain	27.6 (± 30.9)	25	34.0 (± 31.1)	0.286	17	30.4 (± 29.3)	0.955
Dyspnoea	18.7 (± 27.3)	23	23.2 (± 28.5)	0.292	17	25.5 (± 29.2)	0.372
Insomnia	27.6 (± 33.1)	25	53.3 (± 35.3)	< 0.007^c^	17	52.9 (± 39.7)	0.011^c^
Appetite loss	10.1 (± 23.3)	25	22.7 (± 30.9)	0.029^a^	16	12.5 (± 20.0)	0.736
Constipation	9.6 (± 22.3)	25	37.3 (± 36.9)	< 0.001^c^	17	29.4 (± 32.1)	0.047^c^
Diarrhoe	10.4 (± 22.7)	25	13.3 (± 23.1)	0.270	17	13.7 (± 20.0)	0.258
Financial difficulties	11.3 (± 25.0)	25	14.7 (± 25.1)	0.259	17	21.6 (± 30.1)	0.096

*p* values represent the results for an age-matched analysis of our cohort to this norm reference population at baseline and after 2 years

A *p* value of less than 0.05 was considered statistically significant

*N* number of available data, *RT* radiotherapy, *SD* standard deviation

^a^Clinically relevant small difference

^b^Clinically relevant medium difference

^c^Clinically relevant large difference

Baseline symptom scores were significantly higher for fatigue (*p* = 0.003, medium clinical significance), insomnia (*p* < 0.007, large clinical significance), appetite loss (*p* = 0.029, small clinical significance) and constipation (*p* < 0.001, large clinical significance) than those in the normative population. After 2 years, the initially raised scores for fatigue and appetite loss approached normal values, whereas levels for insomnia (*p* = 0.011) and constipation (*p* = 0.047) remained higher than in the general population, each to a large clinical extent.

## Discussion

As oncologic outcomes for patients with cervical and endometrial cancer improve with the use of new diagnosis tools and advancements in imaging and therapeutics, the long-term side effects and quality of life for long-term survivors become all the more important. The prospective phase II APROVE trial analyzed the use of postoperative proton beam therapy for women with endometrial and cervical cancer in the adjuvant setting, aiming to establish a less toxic treatment method. Our study presented encouraging general and gynecologic-specific HRQOL, with improvements in patients’ perception of global quality of life and the maintenance of functioning performance 2 years after treatment.

Multiple components of patient, tumor, treatment, social, psychological and physical characteristics contribute to changes in the quality of life of women with gynecologic cancer at diagnosis and during the course of interdisciplinary treatment. Our results in respect of the comparison of baseline values identified a high degree of impairment in several dimensions of HRQOL for our cancer cohort compared to the norm population. Our mean patient global health status at baseline was 58.0 and thus lower than those reported in cervical and endometrial cancer patients in previous studies, which ranged from 60.3 to 70.6 and partly declined during RT [5, 30–32]. Our cohort did not experience an acute, significant decline in global health status at the end of proton beam therapy and gained an improvement in overall quality of life after 2 years, which was even comparable to the norm reference population group. Controversial results were reported in previous studies, which found a significant decrease in global health status and physical and role functioning during definitive photon radiotherapy for cervical cancer. These impairments improved to baseline levels 3 months after treatment, but remained reduced compared to reference population data [33]. The EMBRACE group for cervical cancer in a definitive setting after radiochemotherapy showed promising oncologic results for local control and a recovery of general quality of life and emotional and social functioning during a longer follow-up of 6 months after treatment, but at the cost of a persistent impairment in cognitive functioning [32].

The findings of prior studies on the post treatment changes of the remaining functional scales are also partly controversial. A prospective study of female gynecologic patients using photon RT by Greimel et al. [34] found global quality of life and emotional functioning to be most impaired at the end of treatment and highly predicted by the extent of upfront surgery and baseline clinical performance status. Contrary to this, our cohort achieved significantly higher emotional and social functioning post treatment and maintained values for the remaining functional levels during the course of proton beam therapy.

Two years after treatment, except for symptoms of fatigue, patients had not recovered from significant changes in impairments of pain, gastrointestinal symptoms and lymphedema, which diminished quality of life, even though patient-reported outcome assessments by physicians only determined low-grade toxicity. Previously reported studies of disease-free patients have observed impaired functioning after photon RT with a larger number of subscales, including higher rates of constipation, diarrhea and menopausal symptoms, which did not significantly lead to impaired quality of life after radiotherapy in our study [30]. While trends toward less fatigue were proven after reducing treatment volumes via the application of brachytherapy compared to EBRT in the PORTEC2 trial [35], no etiological dosimetric correlation could be found for the improvement of fatigue in our cohort of women, who reached levels close to the norm references 2 years after proton beam therapy.

With respect to patient and treatment characteristics, the factors of age, the extent of surgical procedures, tumor stages and other potential factors did not significantly influence HQROL in our study. Contrary to this, intensified treatment concepts with the addition of photon chemoradiation have been shown to have a markedly negative impact on long-term HRQOL and sensory neuropathy in patients with endometrial cancer [5].

Concerning sexual issues, the endometrial subgroup showed improved acute and long-term body image scores and a higher rate of sexual activity at the end of RT, which was not persistent after 24 months for the latter. Contrasting results which showed deterioration during and after the course of treatment have previously been described in women with cervical cancer, when radiotherapy was added to upfront surgery compared to surgery alone [30, 36]. The application of protons did not enable any significant long-term improvements in sexual functioning, sexual interest or vaginal symptoms. This shows that compromised sexual functioning and morbidity still remains a big issue for gynecological cancer survivors in the postoperative setting.

However, the comparisons and conclusions made are mostly focused on the results of wide-spread photon therapy for the treatment of gynecologic cancers, while in the modern era, RT techniques are constantly improving, and the number of more cost-intense proton beam therapy facilities is increasing. In this context, RT techniques were previously reported to have a significant effect on overall quality of life in gynecological cancer patients as well as on functional and symptom scores changes after treatment completion, supporting the benefits of new image-guided intensity-modulated RT compared to 3D-conformal RT [37, 38]. Planning comparison studies in small patient cohorts have proven that intensity-modulated proton beam therapy achieves an excellent target volume coverage and homogeneity [13], and dosimetrically spares organs at risk better than state-of-the-art photon intensity-modulated RT [39–41]. Notably, the applied proton beam delivery techniques (passive vs. active), beam angles and fields, robustness evaluations, patient positioning and localization and inter- and intrafraction variability varied largely throughout the studies.

The beneficial advantage of this theoretical proton dose escalation was reported in planning studies within tumors and extended fields with para-aortic lymph nodes [42, 43]. Compared to photon intensity-modulated radiotherapy, a 32% reduction for the small bowel V(20 Gy) [44] and a 57% reduction for functional bone marrow V(40 Gy) [45] was reported, while dose reductions in the colon and small bowel of up to 50–80% [D(mean)] compared to three-dimensional conformal photon RT were achieved [46]. However, the dosimetric analysis of dosage to the gastrointestinal organs at risk showed no significant correlation to the patient-reported rates of gastrointestinal symptoms in our study. Notably, only one woman in our cohort had an indication for radiotherapy of the para-aortic lymph node region, which may suggest that the benefits and effects of proton beam therapy on extended fields and potentially more meaningful lower doses to the kidneys or small bowel could not be assessed and did not translate into statistically clinical effects. Thus, patient selection criteria for a therapy using protons need to be carefully defined, and the findings may not be generalizable to the overall population of cervical and endometrial cancer patients.

In clinical practice, only a few women (n = 7–11) with gynecological malignancies were reported to be treated with proton beam therapy, but the dosimetric results of statistically significant lower doses to the bowel, bladder and bone marrow are promising, and feasibility studies showed robust beam delivery [14, 15]. However, dose reductions may not necessarily result in clinically observed improvements in the appearance of symptoms or HRQOL and further investigations to determine which gynecologic tumor patients might profit in clinical routine from gastrointestinal toxicity and hematotoxicity reduction from proton beam radiotherapy are urgently needed. In this context, the primary objective of the PROTECT trial [47] will further compare intensity-modulated photon therapy to proton beam therapy for 15 women in each treatment arm for locally advanced cervical cancer in combination with chemotherapy in the definitive setting and will analyze dosages to organs at risk for pelvic bones and the bowel. But recruitment of this prospective phase II trial is still ongoing and results are pending.

In this study, major limitations were caused by the small sample size and the study’s research design with the lack of a control group. Associations of the outcome after proton beam therapy must therefore be interpreted cautiously and further research with a photon radiotherapy control group is needed. Moreover, a partially limited questionnaire response rates might have restricted accurate assessment, especially in terms of sexual issues. Furthermore, patients with cervical and endometrial cancer were observed in one cohort. Even though they followed similar contouring guidelines and consecutively similar resulting planning target volumes, patients’ characteristics and biological responses may have differed. Subgroup analyses must be interpreted cautiously. Further, normative data for the German population is only available for the EORTC QLQ-C30 scales and missing for EN24 and CX24 questionnaires; thus, an age-matched comparison to assess the clinical impact for the latter does not exist and can only be estimated. Psychological effects caused by the application of a new, cost-intense RT method of proton beam therapy, which is not available to all patients, compared to the feeling of receiving standard-of-care photon therapy, can also not be completely ruled out. Concerning geometry-based treated volumes, the conventional CTV to PTV approach, as used in photon therapy, might not be completely applicable for proton beam therapy, in both treatment planning as well as plan evaluation, due to protons range uncertainties. Limitations of statistics include the risk of false-positive conclusion resulting from multiple testing in a small sample size without the use of conservative correction methods, which were not applied to avoid that any interesting correlation, that would be interesting to analyze in a larger phase III trial, may be disregarded. Further, the study protocol chose to censor participants at the time of disease progression instead of 3 months before as well as multiple institutional and standardized CTCAE version 4 symptom scoring systems, which might have led to misleading results.

Nevertheless, proton beam therapy is increasingly implemented in the treatment of women with gynecological cancers and has great potential in administering lower doses to organs at risk and enhanced health recovery during follow-up. Our prospective study represents one of the first cohorts that highlight the impact of postoperative proton radiotherapy on the HRQOL of women with gynecological malignancies and suggests novel, promising results regarding the maintenance of overall quality of life and the impact of only low-grade toxicity. However, to provide adequate health information and patient selection, and for a better understanding of the impact, characterization and treatment of impairments of HRQOL, larger and randomized trials are needed to further confirm the translation of the benefits of proton beam therapy into clinical effects.

## Conclusion

Our study showed that postoperative proton beam therapy for the treatment of patients with cervical and endometrial cancer was well-tolerated and caused only low-grade side effects. The overall quality of life was impaired at baseline, but did not deteriorate during proton beam therapy and even showed promising and enhanced recovery 2 years after radiotherapy to values comparable to those in the norm reference population. Larger and randomized studies are needed to confirm that the benefits of proton beam therapy translate into a clinical effect on HRQOL and to improve patient selection. Furthermore, sexual dysfunction remains an important issue.

## Supplementary Information

**Additional file 1**. **Table S1.** Dose characteristics to organs-at-risk and planning target volume.

## Data Availability

All data generated and analysed during the current study are included in this article.

## Abbreviations

BSO: Bilateral salpingo-oophorectomy
CI: Confidence interval
CT: Computed tomography
CTCAE: Common Terminology Criteria for Adverse Events
FIGO: International Federation of Obstetrics and Gynecology
HRQOL: Health-related quality of life
LNE: Lymph node dissection
RBE: Relative biological effectiveness
RT: Radiotherapy
SD: Standard deviations
TAH: Total abdominal hysterectomy
TLH: Total laparoscopic hysterectomy
