# Supplementary material for: Health-related quality of life and patient-reported symptoms after postoperative proton beam radiotherapy of cervical and endometrial cancer: 2-year results of the prospective phase II APROVE-trial
Source: Radiat Oncol. 2023 Jan 9;18:5. doi: 10.1186/s13014-023-02198-4 (PMC9827629; doi:10.1186/s13014-023-02198-4)
Supplement: Supplementary file 1 — Additional file 1. Table S1. Dose characteristics to organs-at-risk and planning target volume. [file 13014_2023_2198_MOESM1_ESM.docx]

**Table S1.** Dose characteristics to organs-at-risk and planning target volume.

| **Volume** | **median dose (Gy, range or percentage)** | |
| --- | --- | --- |
| **Bladder** | |  |
| Maximum, Gy | | **51.2 (45.9 – 54.1)** |
| Mean, Gy | | **20.2 (15.6 – 29.0)** |
| V30Gy, [%] | | **36.8 (27.9 – 60.5)** |
| V40Gy, [%] | | **29.8 (20.7 – 52.6)** |
| **Rectum** | |  |
| Maximum, Gy | | **50.7 (44.7 – 52.9)** |
| Mean, Gy | | **31.5 (22.5 – 37.6)** |
| V30Gy, [%] | | **63.9 (38.9 – 84.0)** |
| V40Gy, [%] | | **36.3 (20.3 – 58.8)** |
| **sigmoid** | |  |
| Maximum, Gy | | **50.8 (45.5 – 53.2)** |
| Mean, Gy | | **29.2 (15.5 – 37.3)** |
| V40Gy, [cm³] | | **33.2 (8.3 – 128.8)** |
| **small bowel** | |  |
| Maximum, Gy | | **51.2 (43.6 – 53.8)** |
| Mean, Gy | | **6.9 (1.7 – 18.6)** |
| V30Gy, [cm³] | | **140.7 (48.0 – 373.7)** |
| **large bowel** | |  |
| Maximum, Gy | | **46.5 (8.0 – 51.4)** |
| Mean, Gy | | **0.9 (0 – 8.7)** |
| V30Gy, [cm³] | | **8.7 (0 – 102.0)** |
| **PTV** | |  |
| Mean, Gy | | **49.9 (44.5 – 50.2)** |
| D98 | | **46.3 (31.4 – 47.6)** |
| D95 | | **47.6 (37.4 – 48.4)** |
| D2 | | **51.5 (45.9 – 52.6)** |
|  | |  |

PTV: planning target volume. Dx: minimum dose delivered to x% of the PTV.
